# The prevalence of stress among medical students in Syria and its association with social support: a cross-sectional study

**DOI:** 10.1186/s12888-023-04593-3

**Published:** 2023-02-07

**Authors:** Hasan Nabil Al Houri, Sami Jomaa, Douaa Mohammad Nazir Arrouk, Tarek Nassif, Marina J Al Ata Allah, Ahmad Nabil Al Houri, Youssef Latifeh

**Affiliations:** 1 Internal Medicine Department, Damascus University, Damascus, Syria; 2 Internal Medicine Department, Syrian Private University, Damascus, Syria; 3 Faculty of Medicine, Damascus University, Damascus, Syria; 4 Applied Statistics – Quantitative Methods, Damascus University, Damascus, Syria; 5 Department of Psychiatry, Al-Mouwasat University Hospital, Damascus, Syria; 6 Faculty of Medicine, University of Kalamoon, Damascus, Syria; 7 Faculty of Medicine, Syrian Private University, Damascus, Syria; 8 Department of Psychiatry, Faculty of Medicine, Damascus University, Damascus, Syria; 9 Department of Psychiatry, Faculty of Medicine, Syrian Private University, Damascus, Syria

**Keywords:** Stress, Social support, Medical students, Syria

## Abstract

**Introduction:**

Chronic stress can interrupt personal life and cause fatigue, inability to concentrate, and irritability. This study aims to determine the prevalence of stress and its associated factors among medical students in Syria and whether social support could be a protective factor against stress.

**Methods:**

A cross-sectional study was conducted in the faculty of medicine of five Syrian universities. The Medical Student Stressor Questionnaire (MSSQ) was used to evaluate the stress caused by the possible sources of stress. And Social Support Questionnaire – short version (SSQ – short version) was used to assess the social support that medical students received from family, friends, and their fellow medical students using six questions.

**Results:**

A total of 1472 medical students participated in the study. Among the total participants, 671 (45.6%) were males, and 801 (54.4%) were females. The majority of the participators had mild (50.6%; *n* = 745) and moderate (37.0%; *n* = 545) stress levels. Academic-related stressors were the most important cause of stress among undergraduate medical students. Social support was provided equally to both genders, and genders reported the same degree of satisfaction.

**Conclusion:**

Our results emphasize the importance of improving the curricula, providing mental health consultants for students, and paying more attention to the mental health of female students. Finally, students in their clinical years should also receive mental health care, decreasing their duties and providing more self-free time.

## Introduction

Anxiety is the feeling of fear that occurs when faced with stressful situations. It is a normal response when confronted with danger, while fear is always associated with subsequent physical changes, like elevated blood pressure and increased heart rate [1, 2]. Chronic stress, on the other hand, can interrupt personal life and cause fatigue, inability to concentrate, and irritability [3].

Different studies showed higher rates of stress among medical students when compared to the general population [4]. Medical schools aim to produce physicians who can improve public health and provide high levels of patient-centered care; to do so, medical students experience many years of stressful studying and clinical training to become highly competent physicians [4]. In addition, extensive curricula, multiple exams, high academic demands and fear of failure, scarcity of self-free time, work overload, sleep deprivation, poor sleep quality, and facing many career choices and concerns about the future are well-established risk factors for stress among medical students [4, 5]. All these factors might alter the medical students’ emotional and mental health and result in harmful consequences such as deterioration in academic performance, a decline in clinical skills, medical errors, and a lack of empathy toward their patients, in addition to chronic stress, depression, burn-out syndrome, and suicide [4]. In addition, different studies addressed the stress levels among students in public and private universities and how the stress levels could be differed between these groups due to several reasons (i.e., financial considerations, the pressure of living up to the expectations of their families, the pressure of passing exams, the fear of stepping into the real world of medicine) [6, 7].

Syria has an exceptional situation due to the ongoing civil war. Many studies have documented the harmful effects of the Syrian war on mental health [8, 9]. During years of conflict, Syrians suffered unstable and poor living conditions and experienced traumatic events such as violence and torture, the death of loved ones, prolonged deprivation, and extended separation from family members [10]. These factors further adversely affect psychological well-being, manifesting in various common mental disorders (depression, stress, post-traumatic stress disorder, and anxiety).

It is evident that social support from family and colleagues could prevent and even improve stress symptoms [11]. Therefore, recognizing these problems and taking serious action against them is mandatory. In Syria, the limited sources and the lack of information about this problem represent additional contributing factors to poor mental health care services. This study aims to investigate the prevalence of stress and its associated factors among medical students in the five most important universities in the country and whether social support could be a protective factor against stress.

## Methods

The largest and most important public universities are Damascus, Aleppo, Tishreen, and Al Baath. Regarding the fifth university, only three private universities were eligible for the study based on our inclusion criteria; Syrian Private University, University of Kalamoon, and Al Andalus. A random selection was done to choose only one university (the Syrian Private University). Data were collected from March 1st to March 31st, 2022.

We targeted students who were studying at previous universities. Male and female students in their second, third, fourth, or fifth year of college were included. No restriction to age was defined. Students in their 1st year were excluded as this year is a preparatory year, and it determined whether students are going to the medical college based on their academic performance. Students in this year experience different stress-related factors such as massive curricula, the choice they have to make regarding their future, and the surrounding environment. Contrary, students in the sixth year also experience different stress factors as this year is clinical, and students have additional responsibilities toward patients, which is not experienced with their peers in earlier years. Therefore, students in the first and sixth years were excluded; to make a baseline match with all students. Incomplete surveys were also excluded. The G*Power 3.1 software [12] was used to conduct a priori statistical power analysis for sample size estimation, with a two-tailed alpha of 0.05 and a power level of 0.95. Using Cohen’s (1988) criteria, the analysis revealed that a sample size of approximately 595 was needed. Thus, the sample size of 1472 participants included in this study was more than adequate. Participation was voluntary. Students were informed about the purpose of the study and written informed consent was provided by all participants. This study was approved by the Ethical Committee of Damascus University (reference No. 3483) and was conducted in accordance with the Helsinki Declaration as revised in 1989.

### Demographic information

We designed the demographic section to obtain the general characteristics of medical students, which included: gender, year of birth, educational level of parents, and whether one or both of them is a doctor. Financial status, source of income, experience in volunteer work (i.e., volunteer for a community project, sports volunteering, volunteering in hospitals), the current year in college, and grades of the last year were acquired. Financial status was assessed as follows, low: not sufficient to provide basic family needs; medium: sufficient to provide basic family needs; good: to provide basic needs with some luxuries; excellent: provides comfort and luxury. We also asked the participants whether they were living inside or outside Damascus.

### Medical Student Stressor Questionnaire (MSSQ)

Yusoff et al. [13–15] constructed and validated an instrument, the Medical Students Stressor Questionnaire (MSSQ), that uses 40 events to recognize the potential sources of stress among medical students; academic, intrapersonal and interpersonal, teaching and learning, drive and desire, and group activities. Participants were asked to evaluate each of these events by choosing one of five responses: ‘causing no stress at all’, ‘causing mild stresses, ‘causing moderate stress’, ‘causing high stress’ and ‘causing severe stress’. The MSSQ is scored by assigning a value of 0–4 for each of the respective responses. A response of ‘causing no stress at all’ would be scored as 0 and a response of ‘causing severe stresses’ scored as 4. The MSSQ was validated and translated to Arabic [13–15]. We have compared between the multiple groups to detect some of the demographic characteristics that might influence the stress level caused by the potential source of stress.

### Social Support Questionnaire – short version (SSQ – short version)

Arabic version of the social support questionnaire—short version (SSQ6) [16] was used to assess the social support that medical students received from family, friends, and their fellow medical students using six questions. Items were rated by 4-point scales, and scores were summed up to generate a final score ranging from 6 to 24. Higher scores indicate more social support received.

### Statistical analysis

This cross-sectional study measured the perceived stress and social support as dependent variables. Data were collected using Microsoft excel 2016 and included demographic and dependent variables. Responses were processed using the Statistical Package for Social Sciences (SPSS) version 23. Descriptive analysis was conducted for variables, categorical variables were presented as frequency and percentages, and continuous variables were expressed as mean ± standard deviation. A cross-tabulation was performed to determine the differences between the types of Medical Student Stressors (MSS) and demographic variables. We performed a Chi-square T-test to study the association between the stress caused by academic, intrapersonal, and interpersonal, teaching and learning, drive and desire, group activities, and qualitative variables. We validated each subscale and Cronbach's Alpha coefficient for the subscales was calculated. Fisher’s exact test was used whenever any of the expected cells were less than five. The test was two-tailed, and values less than 0.05 were considered statistically significant.

## Results

### Demographic characteristics

The non-response rate of this study was 7.3%. A total of 1472 (92.7%) medical students participated in the study, of which 671 (45.6%) were males, and 801 (54.4%) were females. Regarding year of study, 18.4% of students (*n* = 271) were in their 2nd year, 23.8% (*n* = 351) in the 3rd year, 26.5% (*n* = 390) in the 4th, and 31.3% (*n* = 460) in the 5th year. 14.9% (*n* = 219) of the mothers’ students had an elementary educational level, 21.3% (*n* = 314) had a high school degree, 20.2% (*n* = 297) had institutional degree, and 43.6% (*n* = 642) had a university degree. Whereas 10.5% (*n* = 155) of the fathers’ students had an elementary educational level, 18.9% (*n* = 278) had a high school degree, 12.3% (*n* = 181) had an institutional degree, and 58.3% (*n* = 858) had a university degree (Table 1). All the responders reported some degree of overall stress, but the majority had mild (50.6%; *n* = 745) and moderate (37.0%; *n* = 545) stress levels.

**Table 1:** Shows the demographic characteristics of the participants

Covariates	N (%)
**Gender**	
Male	671 (45.5%)
Female	801 (54.4%)
**Educational level of parents**	
**Mother**	
Elementary	219 (14.9%)
High school	314 (21.3%)
Institute degree	297 (20.2%)
University degree	642 (43.6%)
**Father**	
Elementary	155 (10.5%)
High school	278 (18.9%)
Institute degree	181 (12.3%)
University degree	858 (58.3%)
**One or both parents are doctors**	
No	1277 (86.6%)
Yes	195 (13.2%)
**Financial status**	
Low	81 (5.5%)
Medium	488 (33.2%)
Good	795 (54%)
Excellent	108 (7.3%)
**Voluntary experience if any**	
No	992 (67.4%)
Yes	480 (32.6%)
**Study year**	
2^nd^	271 (18.4%)
3^rd^	351 (23.8%)
4^th^	390 (26.5%)
5^th^	460 (31.3)
**Last semester grade**	
≤ 70%	210 (14.3%)
70—79%	585 (39.7%)
80–89%	590 (40.1%)
≥ 90%	87 (5.9%)
**Place of residence**	
Outside Damascus	976 (66.3%)
Damascus	496 (33.7%)
**Living condition**	
House	1019 (69.2%)
Flat	221 (15%)
University housing	232 (15.8%)
**Source of income**	
Other	4 (0.3%)
The family	1375 (93.4%)
Freelance	93 (6.3%)
**The university**	
Damascus University	556 (37.8%)
University of Aleppo	287 (19.5%)
Tishreen University	295 (20%)
Al-Baath University	123 (8.4%)
Syrian Private University	211 (14.3%)

### Prevalence of stress

We divided the overall prevalence of stress into four categories, low, mild, moderate, and severe; as it was reported in the manual questionnaire [14]. As in Table 2, the overall prevalence of mild stress was observed in more than half of the participants (50.6%; *n* = 745). In addition, the overall prevalence of moderate stress was reported in more than one-third of the participants (37.0%; *n* = 545). Low and severe levels of stress were reported in a minority of participants, 8.8% and 3.6%, respectively (Table 2). The value for Cronbach’s Alpha for medical students’ stressors was α = 0.923.

**Table 2:** Shows the overall prevalence of stress among Syrian medical students

Overall prevalence of stress		
	N	(%)
**Low**	129	8.8
**Mild**	745	50.6
**Moderate**	545	37.0
**Sever**	53	3.6
**Total**	1472	100.0

### Stressors

#### Academic-related stressors (ARS)

Students gave highest ratings for ARS. Females had significant stress caused by ARS compared to males (*p* < 0.05). Severe levels of stress were reported in 17.4% (*n* = 139) of females and 11.3% (*n* = 76) of males, while moderate levels were reported in 53% (*n* = 424) of females and 50.4% (*n* = 338) of males. On the other hand, low and mild levels of ARS were predominant in males, 4.0% (*n* = 27) and 43.3% (*n* = 230), respectively. Students who lived in Damascus city had significantly moderate levels of stress compared to those who lived outside Damascus, 57.9% (*n* = 287) and 48.7% (*n* = 475). A significant relationship was found between last semester's grades and ARS. Severe levels of stress were reported in 18.1% (*n* = 38) of students with a grade of 70% or less, 16.8% (*n* = 89) of those with a grade of 70%-79%, 11.2% (*n* = 66) of those with grades of 80%-89%, and 14.9% (*n* = 13) of students who had a grade above 89% (Table 3). The value for Cronbach’s Alpha for ARS was α = 0.883.

**Table 3:** Shows the prevalence of academic-related stressors

	**ARS**				**χ2**	***P***
**Covariates**	**Low**	**Mild**	**Moderate**	**Severe**		
**Gender**						
Male	27 (4.02%)	230 (34.3%)	338 (50.4%)	76 (11.3%)	18.491	0.0003
Female	19 (2.4%)	219 (27.3%)	424 (52.9%)	139 (17.4%)		
**Financial status**						
Low	1 (1.2%)	18 (22.2%)	40 (49.4%)	22 (27.2%)	16.313	0.061
Medium	21 (4.3%)	156 (32%)	244 (50%)	67 (13.7%)		
Good	22 (2.8%)	244 (30.7%)	418 (52.6%)	111 (14%)		
Excellent	2 (1.9%)	31 (28.7%)	60 (55.6%)	15 (13.9%)		
**Voluntary experience if any**						
No	34 (3.4%)	306 (30.8%)	502 (50.6%)	150 (15.1%)	2.354	0.502
Yes	12 (2.5%)	143 (29.8%)	260 (54.2%)	65 (13.5%)		
**Study year**						
2^nd^	13 (4.8%)	87 (32.1%)	137 (50.6%)	34 (12.5%)	7.887	0.546
3^rd^	6 (1.7%)	102 (29.1%)	185 (52.7%)	58 (16.5%)		
4^th^	11 (2.8%)	119 (30.5%)	207 (53.1%)	53 (13.6%)		
5^th^	16 (3.5%)	141 (30.7%)	233 (50.7%)	70 (15.2%)		
**Last semester grade**						
≤ 70%	5 (2.4%)	42 (20%)	125 (59.5%)	38 (18.1%)	31.106	0.000
70—79%	16 (2.7%)	178 (30.4%)	293 (50.1%)	98 (16.8%)		
80—89%	18 (3.1%)	197 (33.4%)	309 (52.4%)	66 (11.2%)		
≥ 90%	7 (8%)	32 (36.8%)	35 (40.2%)	13 (14.9%)		
**Place of residence**						
Outside Damascus	36 (3.7%)	322 (33%)	475 (48.7%)	143 (14.7%)	14.202	0.003
Damascus	10 (2%)	127 (25.6%)	287 (57.9%)	72 (14.5%)		
**Living condition**						
House	33 (3.2%)	305 (29.9%)	530 (52%)	151 (14.8%)	1.593	0.953
Flat	8 (3.6%)	68 (30.8%)	113 (51.1%)	32 (14.5%)		
University housing	5 (2.2%)	76 (32.8%)	119 (51.3%)	32 (13.8%)		
**The university**						
Damascus University	8 (1.4%)	155 (27.9%)	314 (56.5%)	79 (14.2%)	35.968	0.000
University of Aleppo	14 (4.9%)	110 (38.3%)	131 (45.6%)	32 (11.1%)		
Tishreen University	13 (4.4%)	97 (32.9%)	134 (45.4%)	51 (17.3%)		
Al-Baath University	4 (3.3%)	41 (33.3%)	62 (50.4%)	16 (13%)		
Syrian Private University	7 (3.3%)	46 (21.8%)	121 (57.3%)	37 (17.5%)		

We found no difference between ARS and year of study, financial status, or volunteer experience.

#### Interpersonal and Intrapersonal-related stressors (IRS)

IRS caused significant stress in students of Damascus university (12.2%; *n* = 68) compared to their peers in the other universities, 11.8% in Syrian private university; 9.5% in Tishreen; 9.1% in Aleppo; 7.3% in Al-Baath. Students who lived in Damascus city had significant severe and moderate levels of stress caused by IRS (26.4%; *n* = 131 vs. 10.7%; *n* = 53, respectively) compared to those who lived outside Damascus (22.4%; *n* = 219 vs. 10.6%; *n* = 103). A significant relationship was found between IISs and study year (*p* < 0.5). Severe levels of stress were noted between 14.2% (*n* = 50) of the third-year students and 11.0% (*n* = 34) of the fourth-year students (Table 4). The value for Cronbach’s Alpha for IRS was α = 0.879.

**Table 4:** Shows the prevalence of Interpersonal and Intrapersonal-related stressors

	**IRS**				**χ2**	***P***
**Covariates**	**Low**	**Mild**	**Moderate**	**Severe**		
**Gender**						
Male	228 (33.9)	208 (30.9)	162 (24.1)	73 (10.9)	1.331	0.722
Female	295 (36.8)	235 (29.3)	188 (23.5)	83 (10.4)		
**Financial status**						
Low	24 (29.6%)	20 (24.7%)	24 (29.6%)	13 (16%)	9.174	0.421
Medium	171 (35%)	148 (30.3%)	117 (24%)	52 (10.7%)		
Good	289 (36.4%)	237 (29.8%)	191 (24%)	78 (9.8%)		
Excellent	39 (36.1%)	38 (35.2%)	18 (16.7%)	13 (12%)		
**Voluntary experience if any**						
No	343 (34.6%)	299 (30.1%)	245 (24.7%)	105 (10.6%)	1.865	0.601
Yes	180 (37.5%)	144 (30%)	105 (21.9%)	51 (10.6%)		
**Study year**						
2^nd^	110 (40.6%)	74 (27.3%)	62 (22.9%)	25 (9.2%)	17.071	0.048
3^rd^	102 (29.1%)	105 (29.9%)	94 (26.8%)	50 (14.2%)		
4^th^	136 (34.9%)	123 (31.5%)	88 (22.6%)	43 (11%)		
5^th^	175 (38%)	141 (30.7%)	106 (23%)	38 (8.3%)		
**Last semester grade**						
≤ 70%	63 (30%)	57 (27.1%)	63 (30%)	27 (12.9%)	9.859	0.362
70—79%	218 (37.3%)	176 (30.1%)	127 (21.7%)	64 (10.9%)		
80—89%	209 (35.4%)	182 (30.8%)	142 (24.1%)	57 (9.7%)		
≥ 90%	33 (37.9%)	28 (32.2%)	18 (20.7%)	8 (9.2%)		
**Place of residence**						
Outside Damascus	371 (38%)	283 (29%)	219 (22.4%)	103 (10.6%)	8.375	0.039
Damascus	152 (30.6%)	160 (32.3%)	131 (26.4%)	53 (10.7%)		
**Living condition**						
House	372 (36.5%)	312 (30.6%)	232 (22.8%)	103 (10.1%)	5.0	0.544
Flat	73 (33%)	62 (28.1%)	56 (25.3%)	30 (13.6%)		
University housing	78 (33.6%)	69 (29.7%)	62 (26.7%)	23 (9.9%)		
**The university**						
Damascus University	160 (28.8%)	169 (30.4%)	159 (28.6%)	68 (12.2%)	27.226	0.007
University of Aleppo	113 (39.4%)	82 (28.6%)	66 (23%)	26 (9.1%)		
Tishreen University	119 (40.3%)	93 (31.5%)	55 (18.6%)	28 (9.5%)		
Al-Baath University	47 (38.2%)	42 (34.1%)	25 (20.3%)	9 (7.3%)		
Syrian Private University	84 (39.8%)	57 (27%)	45 (21.3%)	25 (11.8%)		

We found no difference between IRS and gender, volunteer experience, last semester's grade, or financial status.

#### Teaching and Learning-related stressors (TLRS)

More students in Tishreen university (10.8%; *n* = 32) experienced a significant severe degree of stress compared to their peers in the other universities, 9.5% in Syrian private university; 8.1% in Al-Baath; 7.2% in Damascus; 7.0% in Aleppo. However, students in Damascus University reported higher rates of moderate stress (34.2%; *n* = 190) compared to their peers in other universities, 34.1% in Syrian Private University; 31.7% in Al-Baath; 26.1% in Aleppo; 21.0% in Tishreen (Table 5). The value for Cronbach’s Alpha for TLRS was α = 0.817.

**Table 5:** Shows the prevalence of teaching and Learning-related stressors

	**TLRS**				**χ2**	***P***
**Covariates**	**Low**	**Mild**	**Moderate**	**Severe**		
**Gender**						
Male	162 (24.1%)	249 (37.1%)	196 (29.2%)	64 (9.5%)	2.66	0.447
Female	192 (23.9%)	309 (38.6%)	242 (30.2%)	58 (7.2%)		
**Financial status**						
Low	14 (17.3%)	21 (25.9%)	32 (39.5%)	14 (17.3%)	21.696	0.010
Medium	123 (25.2%)	175 (35.9%)	147 (30.1%)	43 (8.8%)		
Good	190 (23.9%)	323 (40.6%)	222 (27.9%)	60 (7.5%)		
Excellent	27 (25%)	39 (36.1%)	37 (34.3%)	5 (4.6%)		
**Voluntary experience if any**						
No	248 (25%)	375 (37.8%)	280 (28.2%)	89 (9%)	5.261	0.154
Yes	106 (22.1%)	183 (38.1%)	158 (32.9%)	33 (6.9%)		
**Study year**						
2^nd^	83 (30.6%)	94 (34.7%)	74 (27.3%)	20 (7.4%)	9.892	0.359
3^rd^	72 (20.5%)	136 (38.7%)	111 (31.6%)	32 (9.1%)		
4^th^	89 (22.8%)	155 (39.7%)	116 (29.7%)	30 (7.7%)		
5^th^	110 (23.9%)	173 (37.6%)	137 (29.8%)	40 (8.7%)		
**Last semester grade**						
≤ 70%	35 (16.7%)	73 (34.8%)	73 (34.8%)	29 (13.8%)	31.327	0.000
70—79%	131 (22.4%)	214 (36.6%)	188 (32.1%)	52 (8.9%)		
80—89%	164 (27.8%)	231 (39.2%)	160 (27.1%)	35 (5.9%)		
≥ 90%	24 (27.6%)	40 (46%)	17 (19.5%)	6 (6.9%)		
**Place of residence**						
Outside Damascus	262 (26.8%)	365 (37.4%)	261 (26.7%)	88 (9%)	20.305	0.000
Damascus	92 (18.5%)	193 (38.9%)	177 (35.7%)	34 (6.9%)		
**Living condition**						
House	236 (23.2%)	389 (38.2%)	304 (29.8%)	90 (8.8%)	6.974	0.323
Flat	49 (22.2%)	82 (37.1%)	73 (33%)	17 (7.7%)		
University housing	69 (29.7%)	87 (37.5%)	61 (26.3%)	15 (6.5%)		
**The university**						
Damascus University	130 (23.4%)	196 (35.3%)	190 (34.2%)	40 (7.2%)	33.872	0.001
University of Aleppo	62 (21.6%)	130 (45.3%)	75 (26.1%)	20 (7%)		
Tishreen University	94 (31.9%)	107 (36.3%)	62 (21%)	32 (10.8%)		
Al-Baath University	26 (21.1%)	48 (39%)	39 (31.7%)	10 (8.1%)		
Syrian Private University	42 (19.9%)	77 (36.5%)	72 (34.1%)	20 (9.5%)		

Students who lived outside Damascus city had a significantly severe degree of TLRS compared to those who lived in Damascus, 9.0% and 6.9%, respectively. Contrary, students who lived in Damascus had higher rates of mild (38.9% vs. 37.4%) and moderate TLRS (35.7% vs. 26.7%) compared to their peers outside Damascus (Table 5).

We found no difference between TLRS and gender, volunteer experience, or year of study.

#### Social-related stressors (SRS)

Severe levels of stress were reported in 2.7% (*n* = 22) of females and 1.8% (*n* = 12) of males, while mild levels were reported in 51.8% (*n* = 415) of females and in 45.5% (*n* = 305) of males. Students in Tishreen university had significant levels of severe stress (3.1%; *p* < 0.05) compared to their peers in the other universities, while students of Aleppo University had the lowest prevalence of severe stress (1.0%). Living outside Damascus had a greater impact on SRS; students who lived outside Damascus city had significant levels of low, mild, and severe stress, 26.8% (*n* = 262), 49.0% (*n* = 478), and 2.7%(*n* = 26), respectively. On the other hand, living in Damascus was associated with moderate levels of stress (28.0%; *n* = 139).

We found no difference between SRS and volunteer experience, last semester grade, year of study, or financial status (Table 6). The value for Cronbach’s Alpha for SRS was α = 0.648.

**Table 6:** Shows the prevalence of Social-related stressors

	**SRS**				**χ2**	***P***
**Covariates**	**Low**	**Mild**	**Moderate**	**Severe**		
**Gender**						
Male	190 (28.3%)	305 (45.5%)	164 (24.4%)	12 (1.8%)	9.935	0.019
Female	179 (22.3%)	415 (51.8%)	185 (23.1%)	22 (2.7%)		
**Financial status**						
Low	15 (18.5%)	39 (48.1%)	23 (28.4%)	4 (4.9%)	11.428	0.237
Medium	126 (25.8%)	227 (46.5%)	120 (24.6%)	15 (3.1%)		
Good	204 (25.7%)	398 (50.1%)	178 (22.4%)	15 (1.9%)		
Excellent	24 (22.2%)	56 (51.9%)	28 (25.9%)			
**Voluntary experience if any**						
No	252 (25.4%)	466 (47%)	247 (24.9%)	27 (2.7%)	6.523	0.089
Yes	117 (24.4%)	254 (52.9%)	102 (21.3%)	7 (1.5%)		
**Study year**						
2^nd^	79 (29.2%)	127 (46.9%)	61 (22.5%)	4 (1.5%)	13.273	0.151
3^rd^	83 (23.6%)	162 (46.2%)	93 (26.5%)	13 (3.7%)		
4^th^	94 (24.1%)	207 (53.1%)	79 (20.3%)	10 (2.6%)		
5^th^	113 (24.6%)	224 (48.7%)	116 (25.2%)	7 (1.5%)		
**Last semester grade**						
≤ 70%	39 (18.6%)	103 (49%)	62 (29.5%)	6 (2.9%)	16.015	0.065
70—79%	144 (24.6%)	295 (50.4%)	128 (21.9%)	18 (3.1%)		
80—89%	159 (26.9%)	285 (48.3%)	139 (23.6%)	7 (1.2%)		
≥ 90%	27 (31%)	37 (42.5%)	20 (23%)	3 (3.4%)		
**Place of residence**						
Outside Damascus	262 (26.8%)	478 (49%)	210 (21.5%)	26 (2.7%)	11.096	0.011
Damascus	107 (21.6%)	242 (48.8%)	139 (28%)	8 (1.6%)		
**Living condition**						
House	257 (25.2%)	500 (49.1%)	242 (23.7%)	20 (2%)	7.8	0.253
Flat	63 (28.5%)	95 (43%)	55 (24.9%)	8 (3.6%)		
University housing	49 (21.1%)	125 (53.9%)	52 (22.4%)	6 (2.6%)		
**The university**						
Damascus University	116 (20.9%)	275 (49.5%)	152 (27.3%)	13 (2.3%)	26.498	0.007
University of Aleppo	87 (30.3%)	143 (49.8%)	54 (18.8%)	3 (1%)		
Tishreen University	84 (28.5%)	150 (50.8%)	52 (17.6%)	9 (3.1%)		
Al-Baath University	35 (28.5%)	52 (42.3%)	33 (26.8%)	3 (2.4%)		
Syrian Private University	47 (22.3%)	100 (47.4%)	58 (27.5%)	6 (2.8%)		

#### Drive and Desire-related stressors (DRS)

Drive and desire-related stressors were the least stress-inducing factor among medical students. Higher levels of severe stress were reported in among Tishreen and Al-Baath students, 6.8% and 6.5%, respectively. Students who had grades of ≤ 70% reported higher levels of stress (11.4%), while those who had grades between 70%-79% and 80%-89% reported lower levels of severe stress, 6.3% and 3.2%, respectively. Students who had volunteer experience had a lower prevalence of moderate (16%) and severe (3.1%) stress compared to those who did not, 18.0% and 6.9%, respectively (Table 7). The value for Cronbach’s Alpha for DRS was α = 0.552.

**Table 7:** Shows the prevalence of Drive and Desire-related stressors

	**DRS**				**χ2**	***P***
**Covariates**	Low	Mild	Moderate	Severe		
**Gender**						
Male	307 (45.8%)	193 (28.8%)	135 (20.1%)	36 (5.4%)	6.46	0.091
Female	393 (49.1%)	240 (29.9%)	121 (15.1%)	47 (5.9%)		
**Financial status**						
Low	24 (29.6%)	29 (35.8%)	21 (25.9%)	7 (8.6%)	34.922	0.000
Medium	200 (41%)	157 (32.2%)	91 (18.6%)	40 (8.2%)		
Good	419 (52.7%)	217 (27.3%)	127 (16%)	32 (4%)		
Excellent	57 (52.8%)	30 (27.8%)	17 (15.7%)	4 (3.7%)		
**Voluntary experience if any**						
**No**	456 (46%)	289 (29.1%)	179 (18%)	68 (6.9%)	10.420	0.015
**Yes**	244 (50.8%)	144 (30%)	77 (16%)	15 (3.1%)		
**Study year**						
2^nd^	148 (54.6%)	78 (28.8%)	33 (12.2%)	12 (4.4%)	13.552	0.139
3^rd^	165 (47%)	101 (28.8%)	63 (17.9%)	22 (6.3%)		
4^th^	169 (43.3%)	114 (29.2%)	83 (21.3%)	24 (6.2%)		
5^th^	218 (47.4%)	140 (30.4%)	77 (16.7%)	25 (5.4%)		
**Last semester grade**						
≤ 70%	78 (37.1%)	64 (30.5%)	44 (21%)	24 (11.4%)	57.205	0.000
70—79%	245 (41.9%)	186 (31.8%)	117 (20%)	37 (6.3%)		
80—89%	318 (53.9%)	162 (27.5%)	91 (15.4%)	19 (3.2%)		
≥ 90%	59 (67.8%)	21 (24.1%)	4 (4.6%)	3 (3.4%)		
**Place of residence**						
Outside Damascus	468 (48%)	281 (28.8%)	167 (17.1%)	60 (6.1%)	1.942	0.585
Damascus	232 (46.8%)	152 (30.6%)	89 (17.9%)	23 (4.6%)		
**Living condition**						
House	485 (47.6%)	300 (29.4%)	180 (17.7%)	54 (5.3%)	6.503	0.369
Flat	93 (42.1%)	69 (31.2%)	44 (19.9%)	15 (6.8%)		
University housing	122 (52.6%)	64 (27.6%)	32 (13.8%)	14 (6%)		
**The university**						
Damascus University	253 (45.5%)	169 (30.4%)	103 (18.5%)	31 (5.6%)	10.063	0.610
University of Aleppo	128 (44.6%)	93 (32.4%)	54 (18.8%)	12 (4.2%)		
Tishreen University	150 (50.8%)	83 (28.1%)	42 (14.2%)	20 (6.8%)		
Al-Baath University	58 (47.2%)	36 (29.3%)	21 (17.1%)	8 (6.5%)		
Syrian Private University	111 (52.6%)	52 (24.6%)	36 (17.1%)	12 (5.7%)		

We found no difference between DRS and gender, place of residence, or year of study.

#### Group Activities-related stressors (GARS)

Higher levels of severe stress were reported in among Tishreen and Al-Baath students, 10.8% and 8.9%, respectively. Females had significant levels of mild, moderate, and severe stress when compared to males. Students who had volunteer experience had a lower prevalence of mild (36.0%) and severe (5.4%) stress compared to those who did not, 41.2% and 8.7%, respectively (Table 8). The value for Cronbach’s Alpha for GARS was α = 0.728.

**Table 8:** Shows the prevalence of Group Activities-related stressors

	**GARS**				**χ2**	***P***
**Covariates**	**Low**	**Mild**	**Moderate**	**Severe**		
**Gender**						
Male	223 (33.2%)	261 (38.9%)	146 (21.8%)	41 (6.1%)	12.07	0.007
Female	208 (25.9%)	321 (40.1%)	201 (25.1%)	71 (8.9%)		
**Financial status**						
Low	16 (19.8%)	28 (34.6%)	24 (29.6%)	13 (16%)	18.113	0.034
Medium	134 (27.5%)	210 (43%)	108 (22.1%)	36 (7.4%)		
Good	243 (30.6%)	303 (38.1%)	191 (24%)	58 (7.3%)		
Excellent	38 (35.2%)	41 (38%)	24 (22.2%)	5 (4.6%)		
**Voluntary experience if any**						
No	268 (27%)	409 (41.2%)	229 (23.1%)	86 (8.7%)	12.333	0.006
Yes	163 (34%)	173 (36%)	118 (24.6%)	26 (5.4%)		
**Study year**						
2^nd^	85 (31.4%)	104 (38.4%)	62 (22.9%)	20 (7.4%)	5.464	0.792
3^rd^	91 (25.9%)	155 (44.2%)	79 (22.5%)	26 (7.4%)		
4^th^	121 (31%)	149 (38.2%)	91 (23.3%)	29 (7.4%)		
5^th^	134 (29.1%)	174 (37.8%)	115 (25%)	37 (8%)		
**Last semester grade**						
≤ 70%	50 (23.8%)	88 (41.9%)	54 (25.7%)	18 (8.6%)	13.202	0.154
70—79%	157 (26.8%)	233 (39.8%)	144 (24.6%)	51 (8.7%)		
80—89%	190 (32.2%)	231 (39.2%)	130 (22%)	39 (6.6%)		
≥ 90%	34 (39.1%)	30 (34.5%)	19 (21.8%)	4 (4.6%)		
**Place of residence**						
Outside Damascus	297 (30.4%)	377 (38.6%)	226 (23.2%)	76 (7.8%)	2.252	0.522
Damascus	134 (27%)	205 (41.3%)	121 (24.4%)	36 (7.3%)		
**Living condition**						
House	298 (29.2%)	400 (39.3%)	244 (23.9%)	77 (7.6%)	0.467	0.998
Flat	65 (29.4%)	87 (39.4%)	51 (23.1%)	18 (8.1%)		
University housing	68 (29.3%)	95 (40.9%)	52 (22.4%)	17 (7.3%)		
**The university**						
Damascus University	153 (27.5%)	224 (40.3%)	142 (25.5%)	37 (6.7%)	10.914	0.536
University of Aleppo	87 (30.3%)	120 (41.8%)	62 (21.6%)	18 (6.3%)		
Tishreen University	86 (29.2%)	106 (35.9%)	71 (24.1%)	32 (10.8%)		
Al-Baath University	40 (32.5%)	46 (37.4%)	26 (21.1%)	11 (8.9%)		
Syrian Private University	65 (30.8%)	86 (40.8%)	46 (21.8%)	14 (6.6%)		

We found no difference between GARS and place of residence, year of study, or last semester grade.

### Social support

Social support was provided equally to both genders, with a mean of 5 persons for each; thus, no statistical difference was found between gender and the perceived support. When asked about their satisfaction regarding the perceived support, both genders reported a degree of 3 out of 5. Students of the five universities had support from an average of five people. Damascus University and Syrian Private University reported the highest degree of satisfaction 3.5 out of 5, while students at the other universities reported a degree of satisfaction of 3 out of 5 (Table 9).

**Table 9:** Shows the scores of the perceived social support and the associated satisfaction degree

Covariates	SSQ Number Score		SSQ Satisfaction score
	Mod (%)	M (SD)	M (SD)
**Gender**			
Male	5 (18.5%)	5 (1.94)	3.31 (1.04)
Female	6 (21.1%)	5 (1.78)	3.3 (1.02)
**Financial status**			
Low	4 (21%)	5 (1.97)	3.23 (1.24)
Medium	5 (19.1%)	5 (1.94)	3.1 (1.09)
Good	5 (20.8%)	5 (1.77)	3.4 (0.95)
Excellent	6 (25%)	5 (1.82)	3.64 (0.98)
**Voluntary experience if any**			
No	5 (19%)	5 (1.87)	3.34 (1.04)
Yes	5 (20.6)	5 (1.84)	3.23 (1.01)
**Study year**			
2^nd^	6 (19.9%)	5 (1.92)	3.08 (1)
3^rd^	4 (17.7%)	5 (1.92)	3.51 (1.03)
4^th^	5 (21.3%)	5 (1.8)	3.3 (1)
5^th^	5 (21.5%)	5 (1.81)	3.29 (1.03)
**Last semester grade**			
≤ 70%	5 (23.8%)	5 (1.93)	3.29 (1.07)
70—79%	5 (19.7%)	5 (1.84)	3.3 (1.03)
80—89%	6 (20.7%)	5 (1.82)	3.34 (0.99)
≥ 90%	6 (19.5%)	5 (1.95)	3.16 (1.14)
**Place of residence**			
Outside Damascus	6 (19.1%)	5 (1.9)	3.2 (1.04)
Damascus	5 (21.1%)	5 (1.74)	3.52 (0.96)
**Living condition**			
House	5 (19.1%)	5 (1.86)	3.27 (1.03)
Flat	6 (22.6%)	5 (1.79)	3.32 (0.98)
University housing	5 (22%)	5 (1.91)	3.46 (1.03)
**The university**			
Damascus University	4 (19.8%)	5 (1.77)	3.56 (1)
University of Aleppo	5 (20.2%)	5 (1.87)	3.01 (0.99)
Tishreen University	6 (19.7%)	5 (1.86)	3.11 (0.95)
Al-Baath University	5, 6 (20.3%)	5 (1.99)	3.15 (1.11)
Syrian Private University	5 (19.9%)	5 (1.93)	3.41 (1.04)

No significant correlation between stressors and received social support was found (Table 10).

**Table 10:** Shows the correlation between stressors and social support among medical students

		**MSSQ**	**SSQ SATISFACTION SCORE**	**SSQ NUMBER SCORE**
**MSSQ**	Pearson Correlation	1	0.042	-0.029
	Sig. (2-tailed)		0.105	0.259
	N	1472	1472	1472

Correlation is significant at the 0.01 level (2-tailed)

## Discussion

Although everyone needs some degree of stress to achieve their best, long-standing, severe stress could alter medical students’ performance and learning abilities and disrupt their skills in taking care of patients. From the beginning of medical school, students endure significant stress, which can motivate them. On the other hand, it may raise feelings of anxiety, ineffectiveness, resentment, and guilt in others [17]. Therefore, social support, especially from family, is required to decrease the stress and improve the well-being of medical students [18]. Cohen et al. defined social support as “a social network’s provision of psychological and material resources intended to benefit an individual’s ability to cope with stress” [19].

Our results showed that 87.6% of Syrian medical students had mild to moderate levels of stress and academic-related stressors were the leading cause of stress among Syrian medical students. A major contributor to this finding is the demanding curricula of medical schools that require students to spend lots of time studying and put them in competition with each other; as a result, students feel overwhelmed and stressed. Eva et al. [7] included 990 Bangladeshi medical students, 357 (36%) of whom were males and 633 females (64%). They reported that academic-related stressors were the leading cause of stress. Another Bulgarian study [11] and many other studies [7, 17, 20–26] have reported the same result. Academic, social, and group-related stressors were more frequent in female students. This could be a normal consequence of our major finding. Females feel more obligated to study, which leads them to spend more time studying compared to their male peers. This would jeopardize their social and group activities and make them feel more stressed when practicing these activities. A study was conducted in the Medical College & Hospital of Basaveshwara reported that ARS, IRS, DRS, and GARS were more predominant in females, whereas TLRS and SRS were more common in males [23]. Most studies concluded that female medical students experience more stress than male colleagues [11, 27–29], which is confirmed by our study results. However, Eva et al. [7] reported no statistical difference in stress levels between both genders. In contrast to many studies. In contrast to many studies [11, 22–24], our results show that the total stress caused by IRS had increased through medical school, 2nd: 18.4%; 3rd: 23.8%; 4th: 26.5%; 5th: 31.3%. However, we found no statistical difference between the study year and the other related sources of stress. Studies show that the percentage of psychological distress was significantly higher in the pre-clinical phase than in the clinical one [11, 22–25]. We believe that the prevalence of stress is higher in clinical years due to scarcity of self-free time, work overload, and feeling more responsible towards patients' health and life. In addition, the fear of not being an outstanding physician and committing medical errors is also considered a factor for stress during the clinical years. This study shows that medical students in public universities suffer more stress than their peers in private ones. Many factors could be responsible for this result. First, students at public universities take their exams for a relatively long period, nearly a month and a half, which make them at risk of experiencing stress rather than other mental health problems. Second, the huge curriculum in public universities also presents contributor to experiencing stress. Finally, public universities have more students on their campuses, and some are experiencing a shortage in learning and teaching equipment, which lessens the ability of students to do their best, and that reflects on their self-confidence and represent an additional source of stress. Eva et al. [7] reported a statistically significant difference (*p* = 0.005) in stress levels between students in public (2.84 ± 0.59) and private (2.73 ± 0.57) universities. Kumar et al. [6] conducted an observational study in public and private medical colleges. They included 312 medical students and also reported that the mean stress score was higher in students of the private university compared to the public university (23.33% vs. 20.61%); the pressure of passing the exam along with the fear of stepping into the real world of medicine were the main reasons for stress among medical students in private universities.

Positive social support from family and colleagues is believed to be a crucial aspect of psychological adjustment that could help buffer the pathogenic effects of stress [30, 31]. Kjeldstadli et al. [32] indicated that medical students who perceived medical school as interfering less with their social and personal lives were psychologically more stable. Many studies have reported that the lack of social support impacts students’ emotion, lowers their learning abilities, and increase the prevalence of anxiety and depression [31, 33–35]. Our study found no significant difference between gender or the students’ university and the perceived support; however, we were unable to find a reasonable explanation for this finding. Thompson et al. [36] reported the same results, whereas Fontana et al. [37] stated that females perceived slightly higher support from their family in comparison with males. Park et al. reported that female students had perceived significant support (*p* < 0.001) [38]. Therefore, active and preventive measures should be brought in by medical schools at the earliest possible because early intervention could buffer the unwanted consequences of psychological distress on medical students’ personal and professional development [24, 39, 40].

Our study has its strengths and limitations. This study is the first to identify stress factors among Syrian medical students. It included students from the major universities in the country with specific criteria in an attempt to make the results more generalizable and accurate. In addition, our study was not limited to a specific stressor; instead, we assessed the six possible causes of stress to come out with transparent results and recommendations. However, some reporting bias may exist as the results were based on the information reported by the students themselves. Another limitation is that we possibly created some report bias when we informed the students about the study aims and how it was designed to measure the stress levels among them. In addition, further studies need to be conducted to compare the stress among medical students and doctors in their residencies. In conclusion, our study found that mild stress is the most frequent among medical students (50.6%), that the most common cause of stress was academic-related stressors and that females were most affected by these factors. This emphasizes the importance of improving the curricula and providing mental health consultants for students. In addition, the female predominance of academic, social, and group-related stressors makes us pay more attention to the mental health of female students. Finally, students in their clinical years should also receive mental health care, decreasing their duties and providing more self-free time.

## Data Availability

The datasets used and/or analyzed during the current study are available from the corresponding author on reasonable request.

## References

[1] Dyrbye LN, Thomas MR, Shanafelt TD (2006). Systematic review of depression, anxiety, and other indicators of psychological distress among U.S. and Canadian medical students. Acad Med.

[2] Wolf TM (1994). Stress, coping and health: enhancing well-being during medical school. Med Educ.

[3] How stress affects your health [https://www.apa.org/topics/stress/health].

[4] Rtbey G, Shumet S, Birhan B, Salelew E (2022). Prevalence of mental distress and associated factors among medical students of University of Gondar, Northwest Ethiopia: a cross-sectional study. BMC Psychiatry.

[5] Paudel K, Adhikari TB, Khanal P, Bhatta R, Paudel R, Bhusal S, Basel P (2022). Sleep quality and its correlates among undergraduate medical students in Nepal: a cross-sectional study. PLOS Global Public Health.

[6] Kumar B, Shah MAA, Kumari R, Kumar A, Kumar J, Tahir A (2019). Depression, anxiety, and stress among final-year medical students. Cureus.

[7] Eva EO, Islam MZ, Mosaddek AS, Rahman MF, Rozario RJ, Iftekhar AF, Ahmed TS, Jahan I, Abubakar AR, Dali WP (2015). Prevalence of stress among medical students: a comparative study between public and private medical schools in Bangladesh. BMC Res Notes.

[8] Carta MG, Moro MF, Bass J (2015). War traumas in the Mediterranean area. Int J Soc Psychiatry.

[9] Quosh C, Eloul L, Ajlani R. Mental health of refugees and displaced persons in Syria and surrounding countries: a systematic review. In: War Trauma Foundation. The Netherlands: ARQ National Psychotrauma Centre; 2013. p. 276–94.

[10] Nguyen TP, Guajardo MGU, Sahle BW, Renzaho AMN, Slewa-Younan S (2022). Prevalence of common mental disorders in adult Syrian refugees resettled in high income Western countries: a systematic review and meta-analysis. BMC Psychiatry.

[11] Georgieva E. Stress and stress factors among medical students in Bulgaria. Albanian J Med Health Sci. 2014;2(2.42):1–19.

[12] Faul F, Erdfelder E, Buchner A, Lang AG (2009). Statistical power analyses using G*Power 3.1: tests for correlation and regression analyses. Behav Res Methods.

[13] Yusoff MSB (1994). A Multicenter Study on Validity of the Medical Student Stressor Questionnaire (MSSQ). Int Med J.

[14] Yusoff MSB, Fuad A. The Medical Student Stressor Questionnaire (MSSQ) Manual. In., edn. 2010. p. 2–21.

[15] Yusoff MSB, Fuad A, Yaacob MJ. The Development and Validity of the Medical Student Stressor Questionnaire (MSSQ). ASEAN J Psychiatry. 2010;11.

[16] Sarason IG, Sarason BR, Shearin EN, Pierce GR (1987). A brief measure of social support: practical and theoretical implications. J Soc Pers Relat.

[17] Dyrbye LN, Thomas MR, Shanafelt TD (2005). Medical student distress: causes, consequences, and proposed solutions. Mayo Clin Proc.

[18] Shao R, He P, Ling B, Tan L, Xu L, Hou Y, Kong L, Yang Y (2020). Prevalence of depression and anxiety and correlations between depression, anxiety, family functioning, social support and coping styles among Chinese medical students. BMC Psychol.

[19] Cohen S (2004). Social relationships and health. Am Psychol.

[20] Kaufman DM, Day V, Mensink D (1996). Stressors in 1st-year medical school: comparison of a conventional and problem-based curriculum. Teach Learn Med.

[21] Kaufman DM, Mensink D, Day V (1998). Stressors in medical school: relation to curriculum format and year of study. Teach Learn Med.

[22] Rock B, Ronald R, Elamparithi T, Zakeena S, Susin M, Sundri R (2017). Prevalance of stress and its risk factors among medical students. Int J Community Med Public Health.

[23] Sm M, NageshRaju G, Singh J, RavishKumar M (2014). A cross -sectional study on the sources and levels of stress among second year undergraduate medical students.

[24] Yee L, Yusoff MSB (2013). Prevalence and sources of stress among medical students in Universiti Sains Malaysia and Universiteit Maastricht. Educ Med J.

[25] Yusoff MS, Abdul Rahim AF, Yaacob MJ (2010). Prevalence and Sources of Stress among Universiti Sains Malaysia Medical Students. Malays J Med Sci.

[26] Yusoff MSB, Yee L, Wei L, Meng L, Bin L, Siong T, Fuad A (2011). A study on stress, stressors and coping strategies among Malaysian medical students. International J Stud Res.

[27] Abdulghani HM, AlKanhal AA, Mahmoud ES, Ponnamperuma GG, Alfaris EA (2011). Stress and its effects on medical students: a cross-sectional study at a college of medicine in Saudi Arabia. J Health Popul Nutr.

[28] Toews JA, Lockyer JM, Dobson DJ, Simpson E, Brownell AK, Brenneis F, MacPherson KM, Cohen GS (1997). Analysis of stress levels among medical students, residents, and graduate students at four Canadian schools of medicine. Acad Med.

[29] Amr M, Hady El Gilany A, El-Hawary A (2008). Does gender predict medical students' stress in mansoura, egypt?. Med Educ Online.

[30] Xiaowen W, Guangping G, Ling Z, Jiarui Z, Xiumin L, Zhaoqin L, Hongzhuan L, Yuyan Y, Liyuan Y, Lin L (2018). Depression and anxiety mediate perceived social support to predict health-related quality of life in pregnant women living with HIV. AIDS Care.

[31] Xu J, Wei Y (2013). Social support as a moderator of the relationship between anxiety and depression: an empirical study with adult survivors of Wenchuan earthquake. PLoS ONE.

[32] Kjeldstadli K, Tyssen R, Finset A, Hem E, Gude T, Gronvold NT, Ekeberg O, Vaglum P (2006). Life satisfaction and resilience in medical school–a six-year longitudinal, nationwide and comparative study. BMC Med Educ.

[33] Aktekin M, Karaman T, Senol YY, Erdem S, Erengin H, Akaydin M (2001). Anxiety, depression and stressful life events among medical students: a prospective study in Antalya, Turkey. Med Educ.

[34] Ball S, Bax A (2002). Self-care in medical education: effectiveness of health-habits interventions for first-year medical students. Acad Med.

[35] Luo Y, Wang H (2009). Correlation research on psychological health impact on nursing students against stress, coping way and social support. Nurs Educ Today.

[36] Thompson G, McBride RB, Hosford CC, Halaas G (2016). Resilience among medical students: the role of coping style and social support. Teach Learn Med.

[37] Fontana MCP, Generoso IP, Sizilio A, Bivanco-Lima D (2020). Burnout syndrome, extracurricular activities and social support among Brazilian internship medical students: a cross-sectional analysis. BMC Med Educ.

[38] Park KH, Kim DH, Kim SK, Yi YH, Jeong JH, Chae J, Hwang J, Roh H (2015). The relationships between empathy, stress and social support among medical students. Int J Med Educ.

[39] Jayarajah U, Lakmal K, Athapathu A, Jayawardena AJ, de Silva V (2020). Validating the Medical Students' Stressor Questionnaire (MSSQ) from a Sri Lankan medical faculty. J Taibah Univ Med Sci.

[40] Saeed AA, Bahnassy AA, Al-Hamdan NA, Almudhaibery FS, Alyahya AZ (2016). Perceived stress and associated factors among medical students. J Family Community Med.

